# Genome-Wide Association Analysis for Resistance to Infectious Pancreatic Necrosis Virus Identifies Candidate Genes Involved in Viral Replication and Immune Response in Rainbow Trout (*Oncorhynchus mykiss*)

**DOI:** 10.1534/g3.119.400463

**Published:** 2019-07-19

**Authors:** Francisco H. Rodríguez, Raúl Flores-Mara, Grazyella M. Yoshida, Agustín Barría, Ana M. Jedlicki, Jean P. Lhorente, Felipe Reyes-López, José M. Yáñez

**Affiliations:** * Facultad de Ciencias Veterinarias y Pecuarias, Universidad de Chile, 8820808, La Pintana, Santiago, Chile; † Facultad de Medicina Veterinaria y Zootecnia, Universidad Nacional del Altiplano, Av. Floral 1153, Puno, Perú; ‡ Escuela Profesional de Medicina Veterinaria y Zootecnia, Facultad de Ciencias de la Salud, Universidad Andina Néstor Cáceres Velásquez, Juliaca, Puno, Perú; § Benchmark Genetics Chile, Puerto Montt, Chile; ** Department of Cell Biology, Physiology and Immunology, Universitat Autònoma de Barcelona, 08193, Barcelona, Spain, and; †† Núcleo Milenio INVASAL, Concepción 4070386, Chile

**Keywords:** Bayes C, *Oncorhynchus mykiss*, Disease resistance, GWAS, QTL, Candidate genes

## Abstract

Infectious pancreatic necrosis (IPN) is a viral disease with considerable negative impact on the rainbow trout (*Oncorhynchus mykiss*) aquaculture industry. The aim of the present work was to detect genomic regions that explain resistance to infectious pancreatic necrosis virus (IPNV) in rainbow trout. A total of 2,278 fish from 58 full-sib families were challenged with IPNV and 768 individuals were genotyped (488 resistant and 280 susceptible), using a 57K SNP panel Axiom, Affymetrix. A genome-wide association study (GWAS) was performed using the phenotypes time to death (TD) and binary survival (BS), along with the genotypes of the challenged fish using a Bayesian model (Bayes C). Heritabilities for resistance to IPNV estimated using genomic information, were 0.53 and 0.82 for TD and BS, respectively. The Bayesian GWAS detected a SNP located on chromosome 5 explaining 19% of the genetic variance for TD. The proximity of Sentrin-specific protease 5 (SENP5) to this SNP makes it a candidate gene for resistance against IPNV. In case of BS, a SNP located on chromosome 23 was detected explaining 9% of the genetic variance. However, the moderate-low proportion of variance explained by the detected marker leads to the conclusion that the incorporation of all genomic information, through genomic selection, would be the most appropriate approach to accelerate genetic progress for the improvement of resistance against IPNV in rainbow trout.

Infectious pancreatic necrosis (IPN) is a highly transmissible disease caused by IPN virus (IPNV) which belongs to the Birnaviridae family, genus Aquabirnavirus (Roberts and Pearson 2005). This virus affects several wild and cultured aquatic organisms. Salmonid species are especially susceptible to IPNV, thus this disease has a great impact on fish farm operations. The mortality levels during an IPN outbreak are influenced by numerous factors; including species, age of the fish, environmental conditions (Dorson and Touchy 1981), and genetic background, which has been proven to confer resistance to some Atlantic salmon (*Salmo salar*) (Guy *et al.* 2006) and rainbow trout (*Oncorhynchus mykiss*) families (Flores-Mara *et al.*, 2017).

Disease resistance represents a broad term defined as the ability of a host to exert some degree of control over the pathogen life cycle (Bishop and Woolliams 2014). From a quantitative genetics point of view, disease resistance in fish can be measured using survival data from either field tests or controlled experimental challenges (Ødegård *et al.* 2011, Yáñez *et al.* 2014). In salmonids it has been possible to determine the presence of significant genetic variation for resistance to bacterial (Gjedrem *et al.* 1991; Gjedrem and Gjøen 1995; Leeds *et al.* 2010; Silverstein *et al.* 2009; Yáñez *et al.* 2013; Yáñez *et al.* 2016; Vallejo *et al.* 2017; Yoshida *et al.* 2018; Barría *et al.* 2018), parasitic (Glover *et al.* 2005; Kolstad *et al.* 2005; Lhorente *et al.* 2012; Yáñez *et al.* 2013; Ødegård *et al.* 2014), and viral diseases (Guy *et al.* 2006; Henryon *et al.* 2005; Ødegård *et al.* 2007). This implies the possibility of improving, through artificial selection, resistance to various diseases in order to enhance disease control strategies in farmed fish.

Prior to include IPNV resistance as part of the breeding goal it is necessary to determine the genetic variance and heritability for the trait. The current dataset comprises animals that were subjected to an IPNV experimental challenge, as previously reported by Yoshida *et al.* (2019). However, these authors focused in estimating genetic parameters and evaluating the accuracy of estimated breeding values using pedigree- and genomic-based information. In contrast, the current work provides novel insights of the genetic architecture and the identification of genomic regions associated to the resistance against IPNV in rainbowtrout. The detection of quantitative trait loci (QTL) is the starting point for determining functional variants involved in quantitative traits. In addition, this information could be used to accelerate the improvement of traits through the application of marker assisted selection (MAS) or genomic selection (GS) (Yáñez *et al.* 2015). The identification of genes that underlie QTL can lead to fundamental knowledge of genetic regulation for disease resistance and host-virus interactions in fish (Moen *et al.*, 2009).

QTL for resistance against various diseases have been determined in salmon and trout (Barría *et al.*, 2018; Kjøglum *et al.*, 2006; Ozaki *et al.*, 2007; Correa *et al.*, 2016; Correa *et al.*, 2015; Moen *et al.*, 2015; Houston *et al.*, 2008; Houston *et al.*, 2012). In Atlantic salmon there is considerable evience for a major QTL for resistance against IPNV in post-smolts, based on data from both field outbreaks (Houston *et al.*, 2008) and experimental challenges (Moen *et al.*, 2009). The discovery of a candidate gene that can bind the virus and successful marker-assited selection for IPNV resistance has also been reported in Atlantic salmon (Moen *et al.*, 2015). However, in rainbow trout there is limited information on the molecular genetic basis of resistance to IPNV. Thus, the aim of the present study was to perform a genome-wide association (GWAS) analysis to determine the genetic architecture for IPNV resistance and to identify potential candidate genes involved in the genetic variation of this trait in rainbow trout.

## Materials And Methods

### Fish

The population used in this study belonged to the breeding population maintained at Aguas Claras S.A. Experimental tests were conducted at the ATC Patagonia Research Center - Aquainnovo (Puerto Montt, Chile). The fish belonged to 58 full-sib families generated from 58 females and 20 males of rainbow trout from the 2014 year-class. For more details on the description about rearing conditions and population management see Flores-Mara *et al.* (2017), Neto *et al.* (2019) and Rodríguez *et al.* (2018).

### Experimental challenge

For a detailed step-by-step protocol for experimental challenge please see Yoshida *et al.* (2019). Briefly, fish at an average age of 154 (SD = 15) days, weighing an average of 2.24g (SD = 0.71) were kept in a single 0.25 m^3^ tank under a fresh water recirculation system. The experimental challenge was performed in two steps; i) intraperitoneal injection of inoculum, at a concentration of 10^7.82^ TCID50/mL, using a quantity of 0.05 mL/fish; and ii) immersion pouring 1.1 L of the inoculum plus 5 L of water into the tank containing 130 L of fresh water, maintained at retained flow at 17° for 4 h. Mortality was individually recorded on a daily basis. At day 63 the experiment was stopped and all surviving animals were killed. Fin clip samples were taken from 768 fish and stored in 98% ethanol at -80° until genomic DNA extraction. The phenotypic data for resistance to IPNV were obtained from a total of 2,278 fish.

### Genotyping

For the protocol used for genomic DNA extraction, genotyping and quality control (QC) please see Yoshida *et al.* (2019). Briefly, each fish was genotyped using a 57K Affimetrix Axiom SNP array developed by Palti *et al.* (2015). The QC included Hardy Weinberg equilibrium, minor allele frequency and call rate for SNPs and samples. The animals and markers which passed the QC were subsequently used for genomic association analysis.

### Genome-wide association analysis (GWAS)

The resistance phenotypes were defined as the time to death (TD), measured in days with values ranging from 1 to 63, depending on the day of death; and the binary survival (BS), recorded as 1 if the individual died during the challenge and 0 if the individual survived until the end of the trial. The Bayes C (Habier *et al.* 2011) analyses were performed using the GS3 software (Legarra *et al.* 2010). A total of 200,000 iterations were used in the Gibbs sampling, with a burn-in period of 50,000 cycles where results were saved every 50 cycles, totaling 4,000 samples. Convergence and autocorrelation were assessed by visual inspection of trace plots of the posterior variance components. The adjusted model can be described, in matrix notation, as follows:

$$y=Xb+Zu+\sum_{i=1}^{n}g_{i}a_{i}\delta_{i}+e$$

where *y* is the vector of phenotypic records for TD or BS; **X** is an incidence matrix of fixed effects; b is the vector of fixed effect (tagging weight as covariate); **Z** is an incidence matrix of polygenic effects; u is a random vector of polygenic effects of all individuals in the pedigree; g_i_ is the vector of the genotypes for the *i^th^* SNP for each animal; a*_i_* is the random allele substitution effect of the *i^th^* SNP, $\delta_{i}$ is an indicator variable (0, 1) sampled from a binomial distribution with such determined parameters that 1% of the markers were included in the model; and *e* is a vector of residual effects.

The proportion of the genetic variance explained by each SNP was calculated according to the following formula: $Vg_{i}=\;(\frac{2p_{i}q_{i}a_{i}^{2}}{\sigma_{g}^{2}}),\;$where $p_{i}$ and $q_{i}$ are the allele frequencies for the i-th SNP; a_i_ is the estimated additive effect of the i-th SNP on the phenotype; and is $\sigma_{g}^{2\;}$the estimate of the polygenic variance (Lee *et al.* 2013).

The association of the SNPs with phenotypes were assessed using Bayes factor (BF), which was calculated as follows: $BF\;=\;(\frac{p}{1-p})/(\frac{\pi }{1-\pi })$, where p is the posterior probability of a SNP to be assigned a non-zero effect and π = 0.99 is the *a priori* probability of a SNP to be included in the analysis (Kass & Raftery 1995; Varona *et al.* 2001).

### Candidate genes

The nucleotide sequences surrounding the top ten SNPs that accounted for the largest proportion of genetic variance for each of the IPNV resistance traits were positioned in the most recent version of the rainbow trout reference genome available from NCBI (GenBank assembly Accession GCA_002163495) (Gao *et al.*, 2018; Pearse *et al.*, 2018) using BLASTx (Basic Local Alignment Search Tool) (Altschul *et al.* 1997). The presence of annotated genes within 1Mb windows surrounding the top ten SNPs was assessed.

### Data availability

Phenotype data and genotypes are available at the online repository Figshare.Supplemental material available at FigShare: https://figshare.com/articles/Untitled_Item/7725668.

## Results

### Experimental challenge and samples

The percentage of accumulated mortality was 13.77%, ranging between 0 to 47.6% for the most and least resistant family, respectively. A selective genotyping strategy was carried out and 768 fish were selected. Table 1 shows the summary statistics of the animals selected for genotyping. For a detailed description about experimental challenge results, please see Yoshida *et al.* (2019).

**Table 1:** Summary statistics for the 768 genotyped fish

Variable	Mean	Standard deviation	Standard error	Minimum	Maximum
**Weight (g)**	2.12	0.74	0.03	0.80	5.80
**Time to death (days)**	51.46	13.98	0.51	13.00	62.00
**Binary survival (0 or 1)**	0.36	0.48	0.02	0.00	1.00

### GWAS and candidate genes

**Figure 1:**
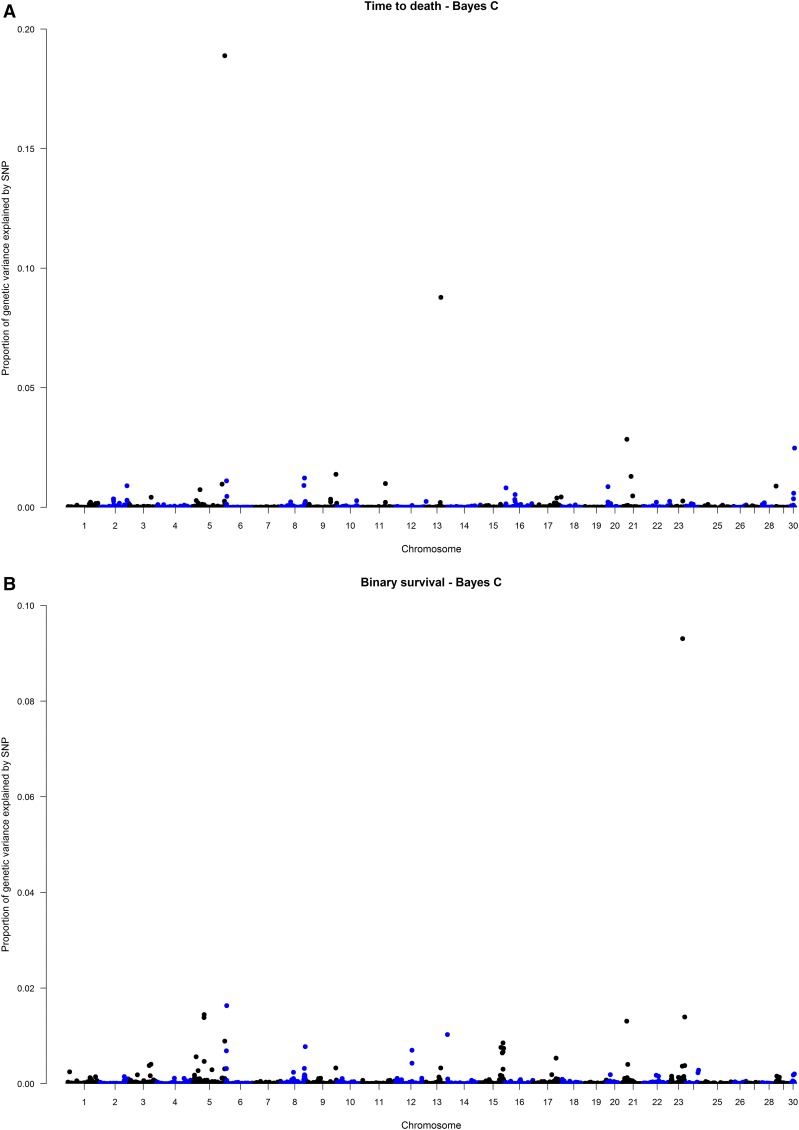
Manhattan plot of variance explained by each SNP using Bayes C method. (A) Time to death. (B) Binary survival.

A total of 721 individuals and 38,296 SNP passed QC. The average number of SNPs per chromosome was 1,277 (SD = 384) and varied between 594 and 1,818. The variance components and heritabilities estimated using genomic information are shown in Table 2. Heritabilities were high for both traits (0.53 ± 0.05 and 0.82 ± 0.03 for TD and BS, respectively). The top SNP for both traits explained 18.89% and 9.31% of the genetic variance for TD and BS, respectively. The Figures 1a and 1b show the manhattan plot for both TD and BS, respectively. According to Vidal *et al.* (2005), a BF from 3 to 20 is suggestive of linkage and BF from 20 to 150 indicates linkage between the SNP and the trait under investigation. In this study, we focused the results and discussion on the SNPs with a BF greater than 20. For TD, the top four SNP that presented a BF greater than 20 are located on chromosomes 5 (AX-89921775), 13 (AX-89964133), 21 (AX-89928391) and 30 (AX-89972475). The sum of these SNPs explained 32.99% of all genetic variance. For the BS trait only the top one SNP presented a BF greater than 20, it is located on chromosome 23 (AX-89938762, Figure 1b) and explained 9.31% of the genetic variance for this trait. The most relevant genes next to the top ten SNPs that explained the highest proportion of genetic variance for each trait are shown in Table 3.

**Table 2:** Mean ± standard deviation of variance components estimated using genome-wide SNPs markers for time to death and binary survival for rainbow trout using Bayes C.

Trait	Total genetic variance^1^	Residual variance	Heritability
Time to death	191.06±139.10	78.60±6.78	0.53±0.05
Binary Survival	4.66±1.05	1.00±0.00	0.82±0.03

1 Correspond to the genetic variance due to markers and the polygenic pedigree-based additive genetic variance.

**Table 3:** Top 10 SNPs associated with resistance to IPNV in rainbow trout identified using Bayes C model.

Marker	Chr^1^	Position (Mb)	BF^2^	Var^3^	Gene^4^^,^^5^(6)
**Time to death**					
AX-89921775	5	86.195	93.824	0.188	Sentrin-specific protease 5 (346.42)
AX-89964133	13	18.398	43.282	0.087	C-C chemokine receptor type 7 (91.96);
					Integrin alpha-11-like (239.47); Secretory phospholipase A2 receptor-like protein (279.15)
AX-89928391	21	40.422	21.698	0.028	NI^7^
AX-89972475	Scaffold	3.313	22.659	0.024	Protein FAM49A ( 228.21)
AX-89919030	9	59.547	15.660	0.013	Protein FAM3A (148.87)
					E3 ubiquitin-protein ligase HUWE1 (373.86)
AX-89929860	21	33.915	14.577	0.013	Interleukin-8 (252.85)
AX-89919605	8	15.655	14.905	0.012	Laminin subunit alpha-4 (403.02)
AX-89964260	6	71.349	13.728	0.011	NI
AX-89954884	11	23.271	14.522	0.010	Integrin beta-1 (498.18)
AX-89955973	5	81.679	13.401	0.009	Dynamin 1 (83.40)
**Binary Survival**					
AX-89938762	23	13.468	22.504	0.093	Apoptosis-stimulating of p53 protein 2-like ( 274.14)
AX-89960378	6	69.996	9.787	0.016	NI
AX-89950201	5	29.774	9.274	0.014	Myosin-IIIb-like (60.73)
					Protein FAM78A-like (418.01)
AX-89951900	23	8.146	9.208	0.014	NI
AX-89944302	5	29.773	8.910	0.013	Myosin-IIIb-like (60.41)
					Protein FAM78A-like (417.69)
AX-89928391	21	40.422	8.618	0.013	NI
AX-89952306	14	0.420	8.264	0.010	NI
AX-89946704	5	86.995	7.395	0.008	Lamin-B2 ( 351.97)
AX-89933583	15	13.021	7.392	0.008	E3 ubiquitin-protein ligase RNF31-like (377.72); Interferon regulatory factor 4-like (337.82); Signalosome complex subunit 5 (428.51)
AX-89962297	8	12.919	7.680	0.007	Unconventional myosin-VI-like (38.04); Collagen alpha-1(XII) chain-like (195.72); Sentrin-specific protease 6 (73.33)

1 Chromosome.

2 Bayes factor.

3 Proportion of variance explained by each individual marker.

4 Suggestive candidate genes located within 1-Mb window.

5 *Oncorhynchus mykiss* used as reference species.

6 Proximity to the SNP (kbp)

7 Not identified

## Discussion

### Experimental challenge and heritability

IPN is one of the main diseases affecting salmonid aquaculture. Our current work was done using data obtained from an experimental challenge to IPNV previously presented by Yoshida *et al.* (2019). Nonetheless, previous authors focused on determining the improvement of genomic prediction accuracies using a single step approach (including genotyped and non-genotyped animals) for the genetic evaluations of resistance to IPNV. The current study focused on providing novel insights of the genetic architecture of IPNV resistance and the genomic regions involved in this trait in a farmed rainbow trout population. The animals used in this study were genotyped using a selective genotyping approach. The allocation of genotyping resources was directed to the most genetically informative progeny (extremely high and low phenotypic values). The selective genotype is a common strategy used to reduce genotyping costs and it has been shown to be highly suitable for QTL detection in genome-wide association studies in livestock and aquaculture species.

The mortality rate in IPNV challenge experiments is highly variable and depends on several factors, including the virus strain, the population evaluated, the size of the fish, environmental conditions, among others. For example, mortality rates over 40% have been reported for Atlantic salmon (Roberts and Pearson 2005; Houston *et al.* 2008). Other authors have reported mortality rates of 70.5% and 77.8% for the same disease and species (Moen *et al.* 2009), while in rainbow trout mortality rates range from 36 to 54% (Ozaki *et al.*,2007).

Some studies have shown significant pedigree-based heritabilities for IPNV resistance in rainbow trout and Atlantic salmon (Flores-Mara *et al.* 2017; Guy *et al.* 2006; Storset *et al.* 2007) using survival time as the trait definition. For instance, in Atlantic salmon, heritability values ranging between 0.26 and 0.55 have been reported for resistance to IPNV, measured as binary survival and analyzed by threshold models (Wetten *et al.* 2007; Kjøglum *et al.* 2008; Guy *et al.* 2009; Gheyas *et al.* 2010; Houston *et al.* 2010). Previous studies have reported pedigree-based heritability values of 0.39 and 0.4 for time to death and 0.35 for binary survival in the same population of IPNV-infected rainbow trout (Flores-Mara *et al.* 2017; Yoshida *et al.* 2019). Moreover, heritability values of 0.24 and 0.25 for day to death and binary survival, respectively, have been reported (Yoshida *et al.* 2019) using a combination of both the pedigree relationship matrix (A) and the genomic matrix (G), also called the H matrix (Aguilar *et al.* 2010). The heritability values calculated in the present study using genomic information are higher than values previously reported in rainbow trout and indicate that selection to improve resistance to IPNV is feasible. The proportion of genetic variance explained by the SNP with the largest effect was larger for trait TD than the SNP identified for trait BS.

### GWAS and candidate genes

Previous studies in Atlantic salmon have determined two significant QTL for resistance against IPNV. The most significant one, explaining 29% and 83% of the phenotypic and genetic variance, respectively, was identified on chromosome 26 (Houston *et al.* 2008; 2012). This QTL was confirmed in an independent Atlantic salmon population from Norway (Moen *et al.* 2009). In rainbow trout, Ozaki *et al.* (2001) found 2 QTL associated with resistance to IPNV using the linkage map elaborated by Sakamoto *et al.* (2000) and based on microsatellite markers. The first QTL was found in linkage group 3 while the second QTL was identified in linkage group 22, each of which explains about 17% of the phenotypic variance (Ozaki *et al.* 2001). The same QTL were confirmed in a subsequent study (Ozaki *et al.* 2007), in which another significant QTL was detected in linkage group 12. Based on the linkage map developed by Palti *et al.* (2012) these significant markers were located on chromosome 14 and 16, respectively (Hu *et al.* 2016).

Some factors could influence the QTL detection, such as the genetic architecture of the traits, the number of animals genotyped and phenotyped, trait’s heritability, linkage disequilibrium (LD) between markers and QTL, and models for QTL detection (Van den Berg *et al.* 2013). The LD is dependent on the demographic history, effective population size, among others, whereas heritability is a property of the trait, population and environmental condition in which the trait was measured (Falconer and Mackay 1996), suggesting that these parameters are intrinsic for a specific population. In our study we did not identify QTL with major effects, similar to those found by Houston *et al.* (2012) and Moen *et al.* (2015) in Atlantic salmon. Moreover, none of the QTL presented here were nearby the known location of the epithelial cadherin gene in the rainbow trout genome. A recent study compared the genomic regions associated to the resistance against a bacterial infection in three salmonid species (*O. kisutch*, *S. salar* and *O. mykiss*), indicating that there is a reduced overlapping between the particular genes involved in the trait among species (Yáñez *et al.* 2019).

The SNP that explained the greatest proportion of genetic variance for TD in the present study (AX-89921775) is located on chromosome 5 (Figure 1a), in a region that contains a gene that encodes *Sentrin-specific protease 5*. In mammals, this protein participates in the SUMO pathway (*small ubiquitin-like modifier*). Its function is mainly related to the activity of isopeptidase (Pinto *et al.* 2012) and the regulation of biological processes that are key to viral replication, including genetic transcription, cell cycle, apoptosis, intracellular and intranuclear trafficking, and protein stability.

Resistance to infection can be the result of a more robust host immune response to the invading agent. In this study we identified SNP located near several genes that encode proteins whose function is to control inflammation as well as other immune-related responses, including the gene that encodes *Secretory phospholipase A2 receptor-like protein* (close to SNP AX-89964133 in chromosome 13). Phospholipases A2 are enzymes released in plasma and extracellular fluids during inflammatory diseases, that induces the release of the pro-inflammatory cytokines TNF-α and IL-6 depending on the concentration of protein (Granata *et al.* 2005).

Thr SNP AX-89933583 (chromosome 15) is associated to BS, which is located in a region that encodes *interferon regulatory factor 4-like (irf4)*. This protein is important in both the regulation of interferons in response to virus infection and the regulation of inducible genes by interferon. In fact, in mammals *irf4* plays a central role in TH cell regulation (Mahnke *et al.* 2016), a fundamental cell-mediated process for the host antiviral response. The SNP AX-89933583 is also located in a gene region that encodes for *signalosome complex subunit 5-like*, that is involved in the regulation of *nuclear factor kappa B* (NF-kB) one of the most relevant transcription factors involved in the control of several pro-inflammatory proteins (Adcock and Caramori 2001).

The activation of the immune response also requires the expression of genes responsible for the activation and recruitment of immune cell populations to the site of infection. In our study, SNP AX-89929860 was located in a gene region that codes for *Interleukin-8* (*IL-8)*, that is a member of the CXC chemokine subfamily directly associated with the pro-inflammatory response because it attracts different immune cell populations including neutrophils, T lymphocytes and basophils (Reyes-Cerpa *et al.* 2012). Importantly, an increased gene expression for *Interleukin-8* was observed in symptomatic IPNV-infected head kidney trout but not in fish with persistent asymptomatic infection (Reyes-Cerpa *et al.* 2014), suggesting its potential effect on the inflammatory response. SNP AX-89954884 is located near *integrin beta 1*; which is a membrane receptor involved in cell adhesion and recognition in a variety of processes including the immune response, indicating a potential role on influencing the pro-inflammatory response on IPNV-infected fish.

Previous reports in Atlantic salmon have shown clear differences in the gene expression pattern between IPNV-susceptible and –resistant phenotypes showing differences in ubiquitin-dependent and apoptosis processes (Reyes-López *et al.* 2015; Robledo *et al.* 2016). In our study two SNPs (AX-89919030; AX-89938762) were located in regions that contain genes that encode for *E3 ubiquitin-protein ligase HUWE1* and *Apoptosis-stimulating of p53 protein 2-like*, respectively. *E3 ubiquitin-protein ligase HUWE1* mediates the proteasomal degradation of target proteins, and the results presented here provide more evidence that ubiquitin-dependent processes could be important factors in IPNV disease resistance. The role of *HUWE1* in proteasomal degradation of target proteins opens the possibility that other processes associated with this function, like vesicular trafficking, could also be involved with susceptibility to IPNV.

The results obtained from this study indicate that the QTL involved in IPNV resistance contribute to a moderate-low proportion of the variance of this trait. From a practical prespective, the implementation of this information in MAS is probably not the most efficient approach (Correa *et al.* 2015).

### Conclusions

This is the first work reporting the detection and position of QTL involved in IPNV resistance in rainbow trout using SNP markers. Resistance to IPNV can be described as a moderately oligogenic trait since there are probably several loci involved; each with a moderate-low effect. Potential candidate genes have been identified close to the associated SNPs, whose biological role in the fish immune response suggest they could be involved in the mechanisms of resistance against IPNV.
